# Antibody–Drug Conjugates and Beyond: Next-Generation Targeted Therapies for Breast Cancer

**DOI:** 10.3390/cancers17243943

**Published:** 2025-12-10

**Authors:** Adil Farooq Wali, Mohamed El-Tanani, Sirajunisa Talath, Syed Arman Rabbani, Imran Rashid Rangraze, Shakta Mani Satyam, Ashot Avagimyan, Karolina Hoffmann, Ioannis Ilias, Sorina Ispas, Maggio Viviana, Anna Paczkowska, Manfredi Rizzo

**Affiliations:** 1 RAK College of Pharmacy, RAK Medical and Health Sciences University, Ras Al Khaimah 11172, United Arab Emirates; sirajunisa@rakmhsu.ac.ae (S.T.); arman@rakmhsu.ac.ae (S.A.R.); manfredi.rizzo@unipa.it (M.R.); 2 RAK College of Medical Sciences, RAK Medical and Health Sciences University, Ras Al Khaimah 11172, United Arab Emirates; imranrashid@rakmhsu.ac.ae (I.R.R.); satyam@rakmhsu.ac.ae (S.M.S.); 3 Department of Anatomical Pathology & Clinical Morphology, Yerevan State Medical University After M. Heratsi, Yerevan 0025, Armenia; avagimyan.cardiology@mail.ru; 4 Isfahan Cardiovascular Research Centre, Cardiovascular Research Institute, Isfahan University of Medical Sciences, Isfahan 81465-1148, Iran; 5 Department and Clinic of Internal Diseases and Metabolic Disorders, Poznan University of Medical Sciences, 61-701 Poznań, Poland; karolinahoffmann@ump.edu.pl; 6 Department of Endocrinology, Diabetes and Metabolism, Elena Venizelou Hospital, GR-11521 Athens, Greece; iliasendo@hippocratio.gr; 7 Department of Anatomy, Faculty of General Medicine, “Ovidius” University, 900470 Constanta, Romania; sorina.ispas@365.univ-ovidius.ro; 8 School of Medicine, PROMISE Department of Health Promotion Sciences Maternal and Infantile Care, Internal Medicine and Medicinal Specialties, University of Palermo, 90133 Palermo, Italy; viviana.maggio01@unipa.it; 9 Department of Pharmacoeconomics and Social Pharmacy, Poznan University of Medical Sciences, 61-701 Poznań, Poland; ananiapaczkowska@ump.edu.pl

**Keywords:** trastuzumab, antibody–drug conjugates, breast cancer, HER2, targeted therapy, precision oncology

## Abstract

Breast cancer is the most common cancer and the most important cause of cancer-related death in females worldwide. Antibody–drug conjugates (ADCs) represent a novel class of targeted therapies that combine the precision of monoclonal antibodies with the potent cell-killing activity of cytotoxic drugs. This review highlights recent mechanistic, technological, and clinical developments of ADCs in breast cancer, including next-generation ADCs beyond those that target HER2 (human epidermal growth factor receptor 2). Authors performed a systematic literature study for ADCs and their structural features, including their components (antibody, linker, and payload) and their therapeutic efficacy. A frame of preclinical research findings and clinical evidence integration of HER2-targeted therapy outcomes in HER2-positive, HER2-low, and triple-negative breast cancer (TNBC) subtypes were presented. Clinical studies of antibody–drug conjugates such as trastuzumab emtansine (T-DM1), trastuzumab deruxtecan (T-DXd), and sacituzumab govitecan have demonstrated significant improvements in progression-free survival and overall survival across diverse breast cancer patient populations. ADCs offer unique advantages in breast cancer therapy by combining the precision of targeted antibodies with the potency of chemotherapy drugs. This allows them to selectively kill cancer cells, overcome resistance, reduce toxicity to healthy tissues, and expand treatment options for difficult subtypes like HER2-low and triple-negative breast cancer. Unlike previous reviews focusing on HER2-targeted ADCs, herein we review exciting ADCs targeting HER3 HER3 (human epidermal growth factor receptor 3) and Nectin-4, as well as the implications of bispecific and immune-stimulatory ADCs in the clinic. Additionally, it features mechanism-based innovations and novel trial data that revolutionize ADC applications in the HER2-low as well as the triple-negative breast cancer subtypes. The advent of ADC is changing precision oncology in breast cancer. With a new design and indications evolving, they are an attractive avenue for bypassing resistance and reducing toxicity and ultimately improving patient outcomes in the molecular subtypes. The present review summarizes recent advancements in antibody–drug conjugates (ADCs) and emerging targeted therapeutic strategies for breast cancer. It covers mechanistic insights, linker–payload innovations, receptor-based targeting approaches, clinical trial progress, and next-generation modalities that extend beyond HER2-directed ADCs. Current challenges, safety profiles, and future opportunities in engineering more selective and effective ADC platforms are also discussed.

## Simple Summary

Breast cancer is the most common cancer in women and remains a leading cause of death worldwide. Traditional treatments like chemotherapy often harm healthy cells, while newer targeted therapies sometimes fail due to resistance or limited effectiveness. Antibody–drug conjugates are an innovative approach that combine the precision of antibodies with the strength of cancer-killing drugs, allowing treatment to be more selective and less toxic. This review explains how these therapies are being improved through better design of their components, such as antibodies, linkers, and drug payloads, and how they are being tested in different types of breast cancer, including those that previously had few treatment options. By highlighting advances beyond current HER2-targeted therapies, this work shows how next-generation strategies may overcome resistance, reduce side effects, and improve survival. These findings provide important guidance for researchers and clinicians working toward more effective cancer care.

## 1. Introduction

Breast cancer remains the most common cancer and leading cause of cancer death in women worldwide. Breast cancer is responsible for an estimated one-sixth of female cancer deaths worldwide and about one-fourth of the total number of new cases [1]. Antibody–drug conjugates (ADCs), a new class of cancer therapeutics, consist of monoclonal antibodies chemically linked to cytotoxic compounds (payload). Antibody–drug conjugates (ADCs) are an innovative class of targeted therapies for breast cancer. They unite the powerful cell-killing ability of chemotherapeutic drugs with the precision of monoclonal antibodies, creating treatments that attack cancer cells more selectively while sparing healthy tissue. Their development reflects a broader shift in oncology toward increasingly refined and personalized targeted therapies [2]. Tamoxifen for estrogen receptor-positive disease and other endocrine therapies that inhibit the hormonal signal were the first major approach to biology-based therapy. Non-selective systemic cytotoxic chemotherapy agent was the cornerstone of early breast cancer therapy.

Two categories of increasingly targeted agents that have been used in the past 20 years due to these continued improvements in molecular subtyping, which classifies breast cancers by subtype, such as luminal A/B, HER2-enriched, triple-negative, and other types, include CDK4/6 inhibitors for hormone receptor-positive disease and immune checkpoint inhibitors in subsets of triple-negative breast cancer (TNBC) [3]. The development of antibody–drug conjugates such as trastuzumab emtansine (T-DM1) and trastuzumab deruxtecan (T-DXd) exemplifies how the precision of antibodies can be combined with highly potent cytotoxic agents to selectively destroy tumour cells while sparing healthy tissues. The heterogeneity of breast cancer requires a multimodal therapeutic strategy combining molecular targeting with optimized drug engineering [4]. These advancements have also been accompanied by advances in linker chemistry, payload heterogeneity, and stimulated effects, which now permit ADCs to be employed in settings other than HER2, including Trop-2 (sacituzumabgovitecan) and HER3 especially with the relapsed/refractory and metastatic state [5]. Furthermore, the field is extending past to immune-engaging ADCs, antibody–radioconjugates, bispecific antibodies, as well as new generations of linker technology such as enhanced tissue penetration, drug stability, and immune modulation [5].

Notably, earlier targeted approaches such as HER2 antibody monotherapy and tyrosine kinase inhibitors often proved ineffective. Antibody–drug conjugates address this therapeutic gap by harnessing distinct cytotoxic mechanisms and leveraging residual antigen expression to achieve more durable antitumour activity. These receptor-dependent mechanisms influence the internalization efficiency, intracellular trafficking, and payload-release kinetics of ADCs. Furthermore, it places these innovations into the context of the changing clinical landscape that includes early stage, resistance evading, and tumour microenvironment-matched therapeutic designs. With ongoing clinical development evaluating newer antigen targets, combination regimens with immunotherapy and the use of ADC for early-stage or minimal residual disease settings are also employed. Next-generation targeted treatments have the potential to transform the landscape of breast cancer, advancing precision oncology toward its ultimate goals of achieving long-term remission, reducing treatment-related toxicity, and improving survival outcomes across diverse patient populations worldwide [6,7]. As the field has expanded, the rationale for next-generation ADCs comprising conjugate antibodies emerged. Recent developments in linker chemistry have significantly improved plasma stability while ensuring efficient intracellular payload release.

## 2. Methodology

To prepare this extensive narrative review, systematic searches of the literature were performed in databases (PubMed, Scopus, and Web of Science). To reflect the evolving field of ADCs in breast cancer, we limited our search to publications spanning January 2010 to August 2025 emphasizing next-generation technologies and clinical applications. Keywords used (1) antibody–drug conjugates (ADC), (2) breast cancer, (3) HER2, (4) HER3, (5) TROP-2, (6) Nectin-4, (7) bispecific ADC, (8) linker technology, (9) targeted therapy. Selection criteria were based on ADC structural components (antibody, linker, payload), mechanistic insights, and clinical outcomes in the HER2-positive, HER2-low, and triple-negative breast cancer subtypes. We prioritized peer-reviewed original research articles, clinical trial reports, and high-impact reviews highlighting new therapeutic approaches or mechanistic insights.

The authors manually screened the references to ensure inclusion of the most recent and clinically relevant studies, while excluding duplicates, conference abstracts, and articles published in languages other than English. The last selection sought a balanced summary of preclinical mechanisms, technological advances, and clinical efficacy data, across distinct molecular subtypes of breast cancer.

To extend beyond conventional ADCs, emerging approaches such as bispecific ADCs, immune-stimulating ADCs, peptide–drug conjugates, and radio-immunoconjugates are being actively explored. These modalities aim to overcome limitations related to antigen heterogeneity, resistance mechanisms, and limited tumour penetration.

## 3. Antibody–Drug Conjugates: Mechanistic Foundations

### 3.1. Structure and Component of ADC

**Figure 1:**
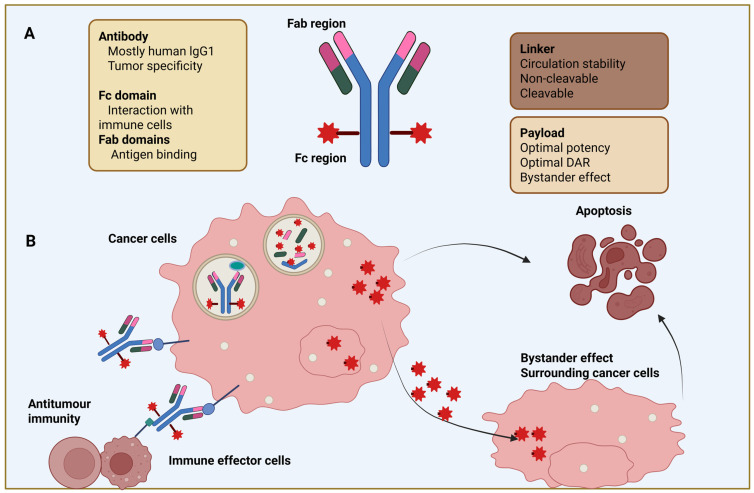
Mechanism and components of antibody–drug conjugates (ADCs) to cancer therapy. Created in BioRender. https://app.biorender.com/illustrations/canvas-beta/68ef5c8ee26f7097780277f0. (**A**) A tumour-targeting monoclonal antibody (most frequently human IgG1, a chemical linker, and a cytotoxic payload antibody with Fab domains for specific binding to an antigen and an Fc domain engaged with immune effector cells. The stability of the linker in circulation is critical and can be cleavable or non-cleavable and this directly impacts the payload release. The payload is designed for high potency, drug-to-antibody ratio (DAR), and bystander effect. (**B**) After binding to vascular-born tumour cell surface antigens, the ADCs become internalized and the payload is released intracellularly, causing apoptosis. This bystander effect enables the payload to impact adjacent cancer cells. The Fc domain also has the potential to harness immune effector cells as well to promote antitumour immunity.

The clinical success in breast cancer depends to a large extent on the exact interplay between their three principle building blocks, namely the monoclonal antibody (MAb), the linker, and the cytotoxic drug. ADCs are highly complex, bioengineered drugs, which can be considered chimeric molecules endowing the potent cytotoxic activity of chemotherapeutic payload with the target-selecting properties of mAbs (Figure 1). An IgG antibody, most often of the IgG1 isotype, serves as the targeting vector [8]. It is selected for its preferable stability, sustained serum half-life, effector functions including ADCC as well as—most significantly—advances in antigen selection, optimized linker technologies, and potent cytotoxic payloads that have collectively strengthened the clinical performance of ADCs and expanded their applicability across different breast cancer subtypes [9].

Major antigens on BCA (breast cancer) cell surface are human epidermal growth factor receptor 2 (HER2), Trop-2, HER3 LIV-1, and Nectin-4, among several others, providing ADCs with receptor binding to attach and initiate internalization [10]. Other key features that have major implications on treatment efficacy and safety, such as binding affinity, endocytosis rate, prevention of entrapment by soluble antigen, or minimization off-tumour targeting, are also scored in the course of antibody selection. The linker is the second key component of an antibody–drug conjugate and represents one of its most technologically innovative features. It serves as the molecular bridge that covalently attaches the cytotoxic payload to the antibody [11].

These findings suggest that variations in antigen expression patterns significantly influence ADCs’ binding, internalization efficiency, and overall therapeutic performance [11]. The two prominent linker technologies in use are the cleavable linkers, which are activated by tumour-specific conditions, such as high concentration of intracellular glutathione (disulphide linkers), protease rich lysosomal compartments (peptide linkers), and acidic pH (hydrazone linkers); meanwhile, non-cleavable linkers require complete catabolism of the antibody through the process of proteolysis in the lysosomes to release the active agent [12].

Several ongoing clinical studies aim to optimize dose regimens and improve safety profiles of ADCs in clinical practice due to their ability to bind selectively to different epitopes and thereby elicit specific immune responses, including antibody-dependent cellular cytotoxicity and complement-dependent cytotoxicity. Increasingly detailed understanding of these mechanisms is facilitating their wider inclusion in medicinal field and their use in therapy of oncological, infectious, and autoimmune diseases. However, as their prevalence increases, so do the adverse effects associated with their use, such as emission of the target antigen due to immunoediting, immune suppression to targeted cells, and resistance to pharmacokinetics of linking detailed knowledge of these mechanisms to clinical outcomes.

### 3.2. Target Antigens in Breast Cancer

The biology of the target antigen is key to ADCs’ design, efficacy, and safety; thus, optimizing the therapeutic potential of ADCs in breast cancer requires a thoughtful selection of target antigens, which are cell-surface proteins functioning as molecular “zip codes” where ADC delivery exclusively refers to cancer cells [13]. High and homogeneous tumour cell surface expression, low normal tissue expression to prevent off-target toxicity, cell surface localisation for accessibility of the antibody part, efficient internalization after binding of the antibody for intracellular payload delivery, and stable expression between disease progression and therapeutic pressure to maintain immune escape are all corresponding features of the ideal ADC target antigen [14]. This receptor, HER-2, is a transmembrane tyrosine kinase that is overexpressed in 15–20% of breast cancers and was associated with aggressive biology and bad prognosis before the era of anti-HER2 therapies emerged one of the most clinically validated targets in breast cancer. Nectin-4 and LIV-1 represent additional emerging antigens with restricted normal tissue expression, reducing off-target toxicity, and expanding ADC applicability. Furthermore, novel immune checkpoint-related antigens such as B7-H4 are under investigation, potentially combining cytotoxicity with immune modulation.

HER2 is particularly appealing for ADC development because of its high and sustained cell surface expression and fast internalization kinetics that enable efficient payload delivery [15]. Examples include the first anti-HER2-targeted ADCs (trastuzumab emtansine (T-DM1)), linking a cleavable linker to the HER2-targeting mAb trastuzumab and combining it with DM1, and trastuzumab deruxtecan (T-DXd), incorporating a potent topoisomerase-I inhibitor payload linked by a cleavable linker, which exhibits superior efficacy in tumours expressing low or heterogeneous levels of HER2 that attributes to its bystander effect. Trophoblast cell surface antigen 2 (TROP-2) is another clinically relevant target of this kind [16]. It is also a calcium-signal transducer and is expressed at low levels under normal conditions in certain epithelial tissues, but it has extensive overexpression in the majority of solid tumours, including many triple-negative and hormone receptor-positive breast cancers where it correlates with increased invasion, proliferation, and worse outcome.

The approval of Sacituzumab govitecan—an ADC juxtaposing an anti-TROP-2 antibody with SN-38, a topoisomerase-I inhibitor, via a hydrolysable linker-highlights TROP-2’s extracellular domain exposure and ubiquity across aggressive subtypes as pivotal for targeted agents alike [16]—provides new options for the treatment of patients with resistant or metastatic triple-negative breast cancer and is more and more under investigation also in hormone receptor-positive disease.

The use of already known targets including HER2, TROP-2, and LIV-1, in addition to more recent antibodies, e.g., HER3, Nectin4, and B7-H4, highlights the potential for certain patients to benefit from dual targeting [4]. It allows ADCs to target the tumour without affecting normal tissue; it also overcomes heterogeneity and resistance observed in various sizes of BC subtypes. The second generation of ADCs (such as bispecific or biparatopic antibodies targeting multiple antigens simultaneously) could be rationalized with respect to the selection of antigen, as other technology becomes available. It is anticipated that this will enhance internalization and decrease therapeutic escape, in addition to enhancing tumour specificity resulting in a colossal shift in breast cancer management across all molecular subtypes [17,18].

SN38 and DX-9851 are also analogues of camptothecin, which is a topoisomerase I inhibitor. SN38 is at least three-fold more potent than irinotecan, the active metabolite of irinotecan [19]. This class of payloads is present in both sacituzumab govitecan (SG) and T-DXd, where the payload has shown to be effective against breast cancer. As an ADC, it is intriguing that irinotecan itself is infrequently used in breast cancer but is beneficial against the disease when its active metabolite (SN38) is delivered. There are two other promising ADCs, aside from T-DXd and SG, involving a topo I inhibitor that is at a more advanced stage of development. These are datopotamabderuxtecan (directed to TROP2) and patritumabderuxtecan (directed to HER3) [20].

Analogous drugs to maytansine DM1 and DM4 are derived from the maytansinoid class of drugs that act by binding to microtubules, leading to arrest in cell cycle during mitosis and death of tumour cells. Although active in subnanomolar ranges, their mechanism of action is by tubulin binding and the same site as that of vinca alkaloids [21]. The first maytansine derivative-based ADC that has been approved by FDA is T-DM1.

### 3.3. Payloads and Linker Technologies

Two key components, the linker technology and the cytotoxic payload, are important for both therapeutic efficacy and clinical success of ADC in breast cancer. These factors combined to play a role in the final potency, selectivity, pharmacokinetics, and stability of ADCs as well as their general therapeutic index [22]. These were a set of obstacles that had to be carefully engineered into next-generation targeted therapeutics. As the drug payload (the attack moiety carried by the antibody) is limited to only a few molecules of each drug, typically 2–8 per antibody, a lethal agent must be developed that represents an ultra-potent class of cytotoxins such as those having sub-nanomolar activity [23]. However, they all have to reach concentrations inside the cell which are high enough to induce death.

In contrast, DNA-damaging agents such as duocarmycins, pyrrolobenzodiazepine (PBD) dimers, calicheamicinsm, and topoisomerase I inhibitors (e.g., deruxtecan and SN-38) interfere with the replication of DNA, leading to double-strand breaks or crosslinking the strands of DNA which triggers stalled growth and programmed cell death due to permanent genomic damage [24]. These payloads are highly cytotoxic and exhibit a potent detrimental effect and have been developed to create HER2-targeted ADCs, such as trastuzumab deruxtecan (T-DXd), in which the monoclonal antibody is linked to deruxtecan through a cleavable peptide linker (Table 1). It has demonstrated significant utility in HER2-low and even HER2-heterogeneous tumours. The detrimental effect, which is facilitated mostly through membrane-permeable payloads from cleavable linkers, takes care of a major resistance mechanism in solid tumours such as breast cancer, where antigen expression is hardly ever homogeneous [25]. This permits diffusion of the cytotoxin into adjacent tumour cells, expressing suboptimal or heterogenenous levels of the antigen.

Next-generation linker technologies are further refining the design of ADCs and they show promise for synergistic immune reactions and tumour-specific activation as well as good plasma stability. These can be hypoxia-activated linkers that respond to the unique metabolic environment of aggressive tumours, or linkers that are cleaved by tumour-specific metalloproteinases [26]. To optimize therapeutic windows, minimize peak toxicities, and prolong anticancer efficacy, select different linkers have been designed to control the rate of payload release. Furthermore, linkers and payloads can be specifically matched for increased efficacy and tolerance. For instance, hydrophilic linkers can enhance the solubility of hydrophobic drugs; self-immolative linkers may enable complete payload release. These findings underscore the importance of linker–payload synergy as follows: an ultra-stable linker without effective release mechanisms limits payload activity and a very potent cytotoxin without a stable, tumour-selective linker may result in non-specific toxicity [26].

As already observed for auristatins, namely monomethyl auristatin E (MMAE) and F (MMAF), are toxic tubulin binders that block the polymerisation of the microtubules. These stable compounds are, however, too toxic for drug administration in their unconjugated format. Auristatin-containing ADCs are represented by disitamabvedotin (target: HER2), brentuximab vedotin (target: CD30), polatuzumabvedotin (target: CD79b), enfortumabvedotin (target: nectin-4), and belantamabmafodotin (target: BCMA) are a few recently approved drugs [27].

**Table 1:** Advancements in linker–payload engineering for ADCs.

S. No.	Innovation Area	Key Advancement	Inference	Reference
1	Cleavable Linkers	Protease-sensitive peptide linkers	Enable selective payload release in lysosomes; improve tumour specificity and reduce systemic toxicity	[28]
2	pH-Sensitive Linkers	Hydrazone-based linkers	Exploit acidic tumour microenvironment; early ADCs showed instability in plasma	[29]
3	Disulfide Linkers	Redox-sensitive cleavage	Utilize elevated glutathione in cancer cells; risk of premature release in circulation	[30]
4	Non-Cleavable Linkers	Lysosomal degradation-dependent	Payload released only after complete antibody breakdown; used in T-DM1 for HER2+ tumours	[31]
5	Self-Immolative Linkers	Cascade payload release	Triggered by enzymatic or chemical cleavage; ensures full payload liberation post-internalization	[32]
6	Hydrophilic Linkers	Solubility-enhancing linkers	Improve pharmacokinetics of hydrophobic payloads; reduce aggregation and off-target effects	[33]
7	High-DAR Engineering	Drug-to-antibody ratio optimization	T-DXd achieves DAR ≈ 8 with stable linker; enhances potency without compromising safety	[34]
8	Payload–Linker Synergy	Rational chemical pairing	Linker chemistry tailored to payload properties; improves release kinetics and tolerability	[35]
9	Bystander Effect Enablement	Membrane-permeable payloads	Cleavable linkers allow diffusion into neighbouring cells; critical for HER2-low and TNBC	[36]
10	Metalloproteinase-Cleavable Linkers	Tumour enzyme-responsive	Target tumours with high protease activity; under exploration for aggressive subtypes	[37]
11	Hypoxia-Activated Linkers	Oxygen-deprivation triggers	Designed for hypoxic tumour cores; selectively release payloads under metabolic stress	[29]
12	Controlled Release Linkers	Tuned payload kinetics	Allow sustained drug delivery; reduce peak toxicity and extend antitumour effect	[38]
13	Dual Payload Strategies	Multi-cytotoxin release	Target multiple resistance pathways; bispecific ADCs under development	[39]
14	Site-Specific Conjugation	Defined antibody attachment sites	Improves ADC homogeneity and stability; reduces variability in DAR	[40]
15	Immune-Stimulatory Payloads	Immunogenic payload integration	Combine cytotoxicity with immune activation; future ADCs may include STING agonists	[41]

### 3.4. Internalization and Cytotoxic Delivery

Ultimately, in breast cancer, the ability of ADCs to effectively target tumour-specific antigens and ensure their internalization has a major impact on their ability to deliver a cytotoxic payload within intracellular compartments capable of causing lethal activity against malignant cells. Thus, the pathway for internalization and trafficking to its cellular environment is a key mechanistic principle that ultimately influences the safety and efficacy profile of these next-generation targeted therapies [42]. Some antigens internalise more slowly and therefore require linker–payload designs that mediate detrimental effect to overcome the lower forms of intracellular delivery, while other antigens undergo faster and more efficient internalization. HER2 remains a highly promising therapeutic target due to its efficient receptor-mediated endocytosis, high surface expression, and well-established role in breast cancer progression of its rapid receptor-mediated endocytosis after ADC binding [40].

The payload diffuses into the cytoplasm or nucleus upon release depending on its molecular target. Microtubule inhibitors, such as the DM1, MMAE, or MMAF that accumulate in cytoplasm and disrupt microtubule formation, are responsible for the G2/M phase arrest at cell cycle progression stage due to mitotic spindle defects, as well as for subsequent activation of intrinsic apoptotic pathways [43]. DNA-damaging agents such as deruxtecan, duocarmycins, SN-38, and pyrrolobenzodiazepine dimers (PBDs) enter the nucleus p53-dependently and -independently to induce single- and double-strand DNA breaks, topoisomerase inhibition, or DNA cross-linking leading to apoptosis. Partial penetrance of certain payloads, deruxtecan and SN-38, for example, which may themselves be membrane-permeable, may be allowed into cells driven by detrimental effect that penetrate cancer cells surrounding DC [44]. This could be an important advantage in breast cancer—a tumour type where intratumoural heterogeneity of target antigens (e.g., HER2-low or heterogeneous HER2 expression) is common. This effect has contributed to the clinical efficacy of ADCs such as sarctuzumabgovitecan and trastuzumab deruxtecan (T-DXd), leading to greater net tumour destruction and reducing the likelihood of immune escape through antigen-negative clones.

Moreover, antigen turnover, recycling dynamics, and shedding of soluble antigens along with intrinsic tumour resistance mechanisms such as lysosomal trafficking defects, overexpression of efflux pumps (e.g., P-glycoprotein), or mutations that disrupt the endocytic pathways themselves have effects on both internalization and cytotoxic delivery efficiency [45]. ADC design strategies now aim to overcome these resistance mechanisms by exploiting the use of biparatopic or bispecific antibodies that cluster receptors and accelerate internalization, or payloads conjugated with novel linkers which release drugs extracellularly in tumour microenvironment for the targeting of non-internalizing antigens. Secondly, a high enough payload must be delivered inside tumour cells, while avoiding premature systemic toxicity and maintaining ADC stability in the circulation, hence site-specific conjugation and optimized drug-to-antibody ratio (DAR) are critical [1,46].

## 4. Clinical Landscape of ADCs in Breast Cancer

### 4.1. Approved ADCs

The clinical landscape for ADCs in breast cancer has transformed dramatically over the past decade. This is since three technology-driven ADCs, trastuzumab emtansine (T-DM1), trastuzumab deruxtecan (T-DXd), and sacituzumabgovitecan have been granted regulatory approval and are widely applied. All of these ADCs are ground-breaking in the field of precision oncology given that they target different tumour subpopulations and are able to reverse resistance to treatment and extend life for patients with advanced stage disease [47]. T-DM1—commonly known as ado-trastuzumab emtansine—is one of the leading treatments for HER2-positive advanced breast cancer and was approved by the FDA in 2013 as the first ADC to target HER2. An agent that has been engineered to covalently link the HER2-targeted monoclonal antibody trastuzumab with the maytansinoid microtubule inhibitor DM1 via a non-cleavable thioether (SMCC) linker, T-DM1 is a stable molecule designed to deliver DM1 selectively to HER2-overexpressing tumour cells while retaining trastuzumab’s mechanisms of action along with its system and downstream immune-mediated effects [48].

In HER2-positive metastatic breast cancer that has been treated, the key EMILIA trial compared T-DM1 with lapatinib plus capecitabine. It was associated with improved overall survival (OS: 30.9 vs. 25.1 months) and progression-free survival (PFS: 9.6 vs. 6.4 months) and a more positive toxicity profile in terms of lesser gastro-intestinal complaints, less alopecia than traditional chemotherapy combinations [49]. For those that progressed on trastuzumab and taxane-based regimens, the results supported T-DM1 as a standard of care in the second-line setting. Further evidence for T-DM1 in a range of disease stages was provided by data from clinical trials such as KATHERINE, which established its use even in early-stage HER2-positive breast cancer as adjuvant treatment for patients with residual invasive disease after neoadjuvant HER2-targeted therapy. In the current trial, T-DM1 reduced the risk of invasive disease recurrence or death by 50% as compared with trastuzumab [50].

Trastuzumab deruxtecan (T-DXd; DS-8201), a HER2-targeted ADC representing the next generation of improved compounds, has transformed the therapeutic prospect of this class in the treatment of both HER2-low as well as HER2-heterogeneous tumours that had no efficient therapies targeting HER2-expressing cells and allowed to build on safety and preliminary efficacy data provided by T-DM1 [51]. T-DXd conjugates trastuzumab to a potent topoisomerase-I inhibitor payload (deruxtecan) through a tumour-selective cleavable tetrapeptide linker. This payload is membrane-permeable, providing potent detrimental effects against neighbouring HER2-low or heterogeneous cancer cells, and has a high drug-to-antibody ratio (DAR ≈ 8). With a median PFS of 28.8 months vs. 6.8 in T-DM1, the pivotal DESTINY-Breast03 trial demonstrated the superiority of T-DXd over T-DM1 for patients with previously treated HER2-positive metastatic breast cancer. This exceptional improvement has again set a new standard for second-line therapy targeting HER2 [52].

Moreover, the field was revolutionized by the DESTINY-Breast04 trial which showed that T-DXd significantly increased OS (23.4 versus 16.8 m) and PFS (9.9 versus 5.1 months) in patients with HER2-low metastatic breast cancer—a fraction of patients long believed to be unsuitable for treatment with HER2-directed therapies aforehand [45]. This made HER2-targeted ADCs applicable as a therapeutic drug to a significantly broader patient population with breast cancer. By demonstrating the clinical relevance of HER2-low as an independent therapeutic subgroup, T-DXd is now a new paradigm-disrupting ADC that can address both high and low HER2-expressing disease in a way that revolutionizes our approach to rating and treating breast cancer [53].

Sacituzumab govitecan (SG) (IMMU-132) is a first-in-class TROP-2-targeting ADC that has transformed the management of TNBC, an aggressive subtype long defined by absent expression of HER2 and hormone receptors, limited treatment options, and poor outcomes. This progress is parallel to the achievements in HER2-targeted ADCs [54]. The active metabolite of irinotecan, the topoisomerase-I inhibitor SN-38, is linked to a humanized anti-TROP-2 monoclonal antibody by the hydrolysable cleavable linker sacituzumabgovitecan. It allows efficient payload delivery inside the TME and drives a robust BK effect, a key aspect for the generally antigen-diverse TNBC. In the pivotal ASCENT trial, sacituzumabgovitecan was shown to be a game-changing therapy for pretreated metastatic TNBC [55]. It presented with higher objective responses rates and a median PFS of 5.6 months compared to the physician’s choice chemotherapy arm of 1.7 months, and OS figures during that phase III study were also higher in nivolumab: 12.1 vs. 6.7 months discussed for previous group allocated to chemotherapy actually selected by physician. These impressive results broadened its role as it received US Food and Drug Administration (FDA) approval in 2020 for metastatic TNBC after at least two prior regimens, followed by expanded approval for hormone receptor-positive, HER2-negative metastatic breast cancer after failure of endocrine therapy and chemotherapy [56]. The success of sacituzumabgovitecan highlights the importance of targeting non-HER2 antigens in breast cancer and that ADCs can treat aggressive subtypes that fail to respond to conventional treatments.

These three approved anticancer drugs are engineered to target distinct therapeutic needs, but in aggregate have shaped a new treatment paradigm for patients with breast cancer. Sacituzumab govitecan provides a targeted alternative for refractory TNBC and subsequently later-line hormone receptor-positive (HR+), HER2- disease; T-DM1 delivers sustained responses and reduced toxicity for the post-trotuzumab patient population; and T-DXd shatters boundaries by demonstrating efficacy in both the HER2-positive and HER2-low subsets of patients with unprecedented PFS and OS benefits [57]. Their clinical success also represents a testimony to how ADC technology has evolved and is a demonstration of continued innovation to overcome the limitations imposed by antigen heterogeneity, drug resistance, and systemic toxicity from the first-generation T-DM1’s non-cleavable linker design through T-DXd’s next-generation high-DAR cleavable linker with detrimental effect and sacituzumab’s tumour microenvironment-responsive linker–payload system [58]. These ADCs not only have efficacy but also generally exhibit better safety profiles than conventional chemotherapy. Typical side effects are manageable haematologic toxicities, gastrointestinal events, and specific problems such as T-DXd-related interstitial lung disease (ILD) which requires close monitoring. These agents are also likely to continue improving survival and quality of life for patients along the entire spectrum of breast cancer by exploring them in earlier stages of disease, neoadjuvant, and adjuvant settings, and in combination with immune checkpoint inhibitors or other targeted agents [59].

The approvals of T-DM1, T-DXd, and sacituzumabgovitecan not only represent major treatment advancements but also reflect the increasing importance of ADCs as important therapeutic players in modern day breast cancer therapy. This may pave the way for next-generation ADCs targeting new antigens, also exploiting dually specific antibodies and cancer immune payload conjugates to address continuing issues of tumour heterogeneity, resistance, and metastasis.

Several clinical trials have defined the evolutionary course of antibody–drug conjugates in advanced breast cancer. The DESTINY-Breast03 trial evaluated the magnitude of improvement in median progression-free survival with trastuzumab deruxtecan versus trastuzumab emtansine. Patients randomized to T-DXd experienced a PFS of 28.8 months, in contrast to just 6.8 months of T-DM1 (HR = 0.33; *p* < 0.001). In relation to HER2-positive disease, T-DXd can be deemed the optimal pharmacotherapeutic option. The ASCENT trial assessed the utility of sacituzumabgovitecan in metastatic triple-negative breast cancer, noting a median PFS of 5.6 months versus 1.7 months with physician’s choice chemotherapy and a median OS of 12.1 vs. 6.7 months, respectively [60]. In connection with pretreated HR-positive, HER2-negative breast cancer, the TROPION-Breast01 trial studied datopotamabderuxtecan with a PFS of 6.9 months, compared to 4.9 months estimated for standard chemotherapy (HR = 0.63; *p* < 0.001). In sum, these landmark studies highlight how next-generation ADCs, those with topoisomerase I inhibitor payloads, and optimal linker chemistry exhibit superior efficacy and manageable toxicity across molecular subtypes.

### 4.2. ADCs in Triple-Negative Breast Cancer (TNBC)

Sacituzumab govitecan and a growing next-generation ADC pipeline directed against diverse tumour antigens and resistance mechanisms have transformed the therapeutic landscape in TNBC, a historically challenging and deadly subtype of breast cancer, from one with little targeted options to one with several promising candidates. TNBC accounts for 15–20% of all breast cancers and is associated with aggressive clinical behaviour, early visceral and central nervous system (CNS) metastasis, high rates of relapse, and poor overall survival (OS), when compared to other subtypes [61]. It is also referred to as negative for estrogen receptor (ER), progesterone receptor (PR), and HER2. Before the advent of poly (ADP-ribose) polymerase inhibitors, standard cytotoxic chemotherapy such as anthracyclines, taxanes, and platinum salts, and more recently immune checkpoint inhibitor for PD-L1-positive disease, were the backbone of TNBC treatment. These treatments offered little benefit beyond modest response rates and no durable control to patients with relapsed or refractory metastatic TNBC.

The intratumoural heterogeneity in TNBC encompasses various subtypes such as basal-like, mesenchymal, and luminal-androgen receptor-positive TNBC, and the lack of well-defined actionable molecular targets further complicated treatment challenges [62]. However, the advent of ADCs has transformed management for TNBC, capitalizing on tumour-specific surface antigens to deliver potent cytotoxic payloads directly to malignant cells and minimize systemic toxicity. Traditional chemotherapy has thus become more effective and well tolerated (Table 2).

**Figure 2:**
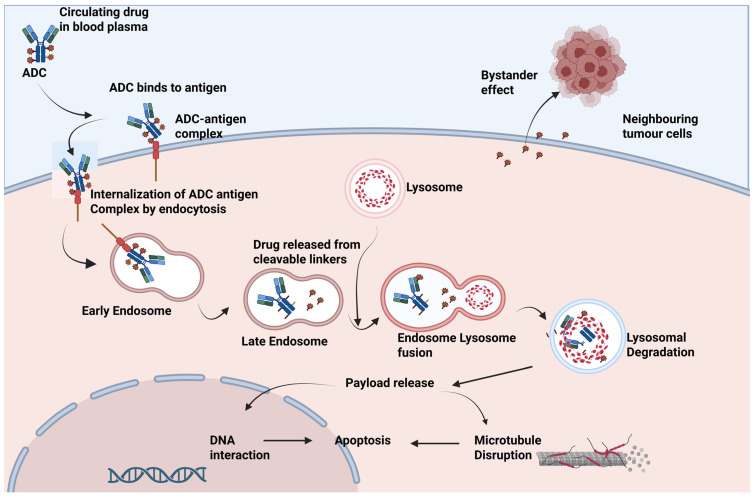
Mechanism of action and intracellular trafficking of ADC. Created in BioRender. https://app.biorender.com/illustrations/68f1ec7e514c3d6665171a4e. Steps are sequentially shown in the figure for mechanism of action of ADCs in tumour cells. ADCs are directed to the bloodstream where they associate and attach to tumour-expressed antigens on the surface of the tumour cell. ADC–antigen complex is taken up through endocytosis and progresses through early and late endosomes. ADC structure contains a cleavable linker that promotes the intracellular re-packaging and subsequent release of the cytotoxic payload following endosomal maturation. ADC is degraded in this fashion, and the released payload continued after fusion of the late endosome with lysosomes. Released drug cytotoxicity occurs through binding to DNA to cause apoptosis and disrupting microtubules. It also demonstrates a detrimental effect whereby the drug released can spread to adjacent tumour cells further increasing therapeutic activity beyond just antigen-positive targets.

The first specific agent that showed benefit in this setting was sacituzumabgovitecan (IMMU-132), a TROP-2-targeting ADC and a first-in-class weapon in the war against TNBC with ADCs (Figure 2). Tromatoblast cell-surface antigen-2, also known as TROP-2, is a transmembrane glycoprotein that has a wide range of expression, ranging from 80 to 90% of TNBC tumours, and little of the most normal tissues, constituting a potential target for therapy. Sacituzumab govitecan links a humanized anti-TROP-2 IgG1κ monoclonal antibody and SN-38, which is the active metabolite of irinotecan and a potent topoisomerase-I inhibitor, with a hydrolysable cleavable linker [63]. This facilitates the efficient release of payloads within lysosomes upon ADC internalization and in the acidic tumour microenvironment. An important feature for overcoming TNBC’s well-known antigen heterogeneity is the membrane-permeable property of SN-38 and its high drug-to-antibody ratio (DAR = 7.6), which facilitate a robust detrimental effect and cytotoxicity in TROP-2-low or -mixed populations of cancer cells nearby. The landmark ASCENT trial (a randomized, phase III study of sacituzumabgovitecan vs. single-agent chemotherapy in patients with pretreated metastatic TNBC who had received at least two prior regimens) demonstrated unprecedented clinical benefits with >5× and a seven-fold increase in ORR (35% vs. 5%), PFS of 1.7 vs. 5.6 months, and OS of 6.7 vs. 12.1 months, respectively [64]. Patients also described improvements in the quality of their lives. These studies led to FDA-accelerated approval in 2020 and full approval in 2021 of sacituzumabgovitecan for patients with metastatic TNBC who previously received two or more prior lines of systemic therapy. The ASCENT trial, which addressed an important unmet need, showed consistent efficacy also in clinically relevant subgroups including those with brain metastases. Treatment-emergent AEs including nausea, diarrhea, and neutropenia were generally of low grade and manageable with common supportive care or dose modification, demonstrating the favourable risk–benefit ratio of this novel anti-TROP-2 ADC [65].

**Table 2:** Therapeutic landscape of ADCs across breast cancer subtypes.

S. No.	Breast Cancer Subtype	ADCs Therapy	Scientific Contribution/Finding	Reference
1	HER2-positive	T-DM1 (ado-trastuzumab emtansine)	Improved OS and PFS vs. lapatinib + capecitabine	[66]
2	HER2-positive	T-DXd (trastuzumab deruxtecan)	Superior efficacy over T-DM1 in second-line setting	[67]
3	HER2-low	T-DXd	First ADC to show benefit in HER2-low tumours	[68]
4	Triple-Negative (TNBC)	Sacituzumab govitecan	Significant OS and PFS improvement in refractory TNBC	[61]
5	HR+/HER2−	Sacituzumab govitecan	Expanded use in endocrine-resistant HR+ disease	[69]
6	HER3-expressing	Patritumabderuxtecan	Promising activity in HER2-low and HER3+ tumours	[70]
7	TROP-2-expressing	Dato-DXd (datopotamabderuxtecan)	Phase 3 signals in TNBC and HR+ disease	[71]
8	Folate receptor alpha	Mirvetuximabsoravtansine	ADCs targeting folate receptor in HR+ tumours	[72]
9	Nectin-4	Enfortumabvedotin	Targeting nectin-4 in TNBC with high internalization	[73]
10	LIV-1	Ladiratuzumabvedotin	Targeting EMT-associated antigen in TNBC	[74]
11	HER2-positive (early-stage)	T-DM1 (adjuvant)	Reduced recurrence risk post-neoadjuvant therapy	[75]
12	HER2-low (heterogeneous)	T-DXd	Bystander effect enables efficacy in low-expression tumours	[76]
13	TNBC with brain metastases	Sacituzumab govitecan	Demonstrated intracranial activity in ASCENT subgroup	[77]
14	HR+/HER2− (late-line)	Dato-DXd	Durable responses in heavily pretreated patients	[78]
15	HER2-positive (resistant)	T-DXd	Effective in trastuzumab-resistant and T-DM1-refractory cases	[70]

### 4.3. Emerging ADCs in Clinical Trials

An ADC pathway that expands the concept of ADCs to novel targets, payload classes, and molecular formats, testing combinations aimed at overcoming resistance mechanisms and leveraging tumour immunobiology, the clinical landscape of emerging ADCs in breast cancer, is highly dynamic at present [79]. Recent studies and trial reports cited significant large success in PFS and OS validating putting the strategy to optimize linker/payload (DXd) chemistry as well as DAR so to elicit bystander activity into antigen heterogenous tumours. Datopotamabderuxtecan, a TROP-2-targeting DXd-based ADC, has demonstrated a strong signal in TNBC and other breast cancer indications.

Novel ADCs are being developed with stable, tumour-responsive linkers, site-specific conjugation, and optimized drug-to-antibody ratios to improve safety and predictability. They also explore new payloads beyond tubulin inhibitors, including DNA-damaging and immune-modulating agents, and bispecific designs to overcome tumour heterogeneity. However, the market still lacks ADCs effective in antigen-low tumours, those with non-tubulin payload classes, constructs engineered for deeper solid-tumour penetration, and safer long-term therapies suitable for maintenance use.

The ongoing Phase 3 TROPION-PanTumour trial in the first-line unselected locally advanced or metastatic TNBC. Numerous important initiatives had been undertaken with regard to advancing earlier-stage assets comprising eight ESMO abstract presentations on lead product candidates, six ESMO abstract submissions of late-breaker data; the highly encouraging results for datopotamab Dato-DXd from the ongoing pivotal study warrant an expansion into first-line treatment experience under the TROPION programme, given that it could offer significant benefits to all patients [80].

**Figure 3:**
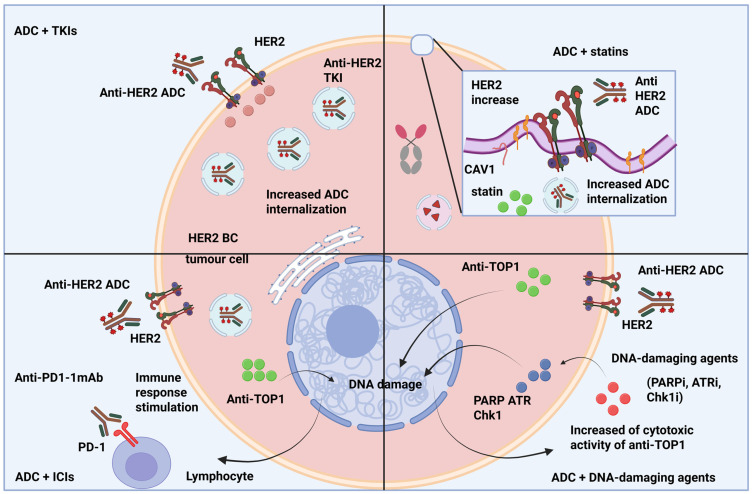
Synergistic strategies to increase the efficacy of antibody–drug conjugates in HER2-positive breast cancer. Created in BioRender. https://app.biorender.com/illustrations/68ef80f2e26f7097782a8109. HER2-positive breast cancer cells are depicted in the main drawing of the figure, including both DNA damage and immune cellular interaction, illustrating the complex integration of molecular and immunological modulation that enhances ADC therapy. Four complementary strategies to enhance the therapeutic benefit of anti-HER2 ADC are illustrated in this figure. Top left: dual TKI usage drives HER2 receptor endocytosis leading to increased intracellular availability of ADC. Top right: statins are known to suppress caveolin-1 (CAV1), which in turn promotes HER2 expression and enhances ADC binding and internalization. Bottom left: immune checkpoint inhibitors (ICIs), known to include anti-PD1 monoclonal antibodies (MoAbs), can infuse immune effector cells after activation, thereby enhancing the ADC-mediated antitumour immunity. Bottom right: TOP1I inhibitors lead to DNA damage resulting in activation of the PARP and Chk1 pathways, which drive tumour sensitivity toward ADC-induced cytotoxicity.

In addition, randomized and regulatory interest is needed for the extraordinary single-agent activity of HER3-targeted DXd ADCs (patritumabderuxtecan/HER3-DXd) observed in heavily pre-treated hormone-receptor-positive, HER2-negative populations (Figure 3). The early cohorts show objective response rates far exceeding historical controls, and the brain-metastasis cohort shows evidence of CNS activity [81].

Ladiratuzumabvedotin (LIV-1–MMAE) is being developed as a monotherapy and in combination with immune checkpoint inhibitors, seeking to maximize potential synergies between these agents and increase response rates in TNBC [82]. The disitamabvedotin (RC48) is also under investigation now in HER2-positive and -low diseases; it is one of the MEK inhibitors/HER2 axis, trametinib/mechanistic target of rapamicin combination beyond the design to treat trastuzumab. It has resulted in encouraging test responses rationalize further development and combination strategies [83].

Mechanistic field expanders are also explored, to further increase tumour selectivity and responses and reduce systemic toxicity. This company is developing bispecific (BIPARATOPIC) ADCs, site-specific conjugates, payload diversification, and TME-activated linkers [84].

Comparing the relative efficacy of antibody–drug conjugates across clinical trials is inherently complicated by differences in study design, eligibility criteria, level of prior therapy exposure, and definitions of trial endpoints. The DESTINY-Breast, ASCENT, and TROPION trials enrolled distinct patient populations understudied with respect to the application of ADCs in the clinical setting—HER2-positive, triple-negative, and HR+/HER2− breast cancers, respectively. As such, direct cross-trial comparison of median PFS or OS without considering these elemental differences is inappropriate. Moreover, the heterogeneity of trial endpoints introduces additional ambiguity to the interpretation of clinical trials. While some evaluate response to therapy based on standard radiologic RECIST criteria, others define PFS as a composite clinical endpoint or utilize investigator assessment. Variability in follow-up duration, interval of response assessment, and statistical cutoff may also affect the apparent efficacy outcomes (Table 3).

In comparison, standardization of key endpoint definitions and efficacy metrics would allow for meaningful comparison and meta-analysis of ADC trials. Similarly, consistent trial endpoints would facilitate cross-study benchmarking and enable a systematic evaluation of evidence for treatment sequencing. Efforts to harmonize definitions of clinical benefit and response gained momentum with the inception of the ESMO Magnitude of Clinical Benefit Scale and progress in FDA-EMA alignment on endpoint criteria. Application of similar standards to ADC research would permit clinicians and regulatory bodies competent in the undefined to gain a more informed perspective on comparative efficacy, safety profiles, and the incidence of resistance across developing classes of ADCs.

### 4.4. Comparative Efficacy

Fam-trastuzumab deruxtecan (T-DXd, ENHERTU) exemplifies this way by pairing a high-potency topoisomerase I inhibitor payload with a cleavable linker, and a high drug-antibody ratio, T-DXd, produced clinically meaningful responses in patients with HER2-low breast cancer and has moved rapidly through pivotal trials, earning regulatory approvals that expanded the HER2 treatment paradigm beyond the binary HER2-positive/HER2-negative split and prompting subsequent label expansions into HR-positive, HER2-low/ultralow metastatic disease; these advances underscore how ADC design can convert “untargetable” biomarker niches into actionable therapeutic opportunities [85]. Parallel to T-DXd, sacituzumabgovitecan (TRODELVY), a Trop-2-directed ADC that delivers SN-38 (the active metabolite of irinotecan), achieved statistically significant survival benefits in heavily pretreated metastatic TNBC and subsequently demonstrated activity in HR-positive/HER2-negative populations, establishing Trop-2 as a broadly relevant ADC target and showing that payload choice and target biology together determine both efficacy and the toxicity profile [71].

Clinically, the ADC era in breast cancer is now characterized by a proliferation of randomized, often head-to-head or benchmarked trials (e.g., DESTINY-series for T-DXd, ASCENT for sacituzumabgovitecan, TROPiCS-02, and others) that have not only improved progression-free and overall survival compared with standard chemotherapies in specific settings but also raised new questions about optimal sequencing, cross-resistance, and patient selection; for instance, DESTINY-Breast trials showed pronounced activity of T-DXd versus T-DM1 in certain HER2-positive populations and supported the expansion into HER2-low disease, while ASCENT demonstrated clinically meaningful benefit for sacituzumabgovitecan in refractory TNBC—findings that have reshaped treatment algorithms and present comparative real-world analyses and academic efforts to define the most effective sequence of ADCs in HER2-negative disease [53].

## 5. Resistance Mechanisms and Biomarker Challenges

### 5.1. Antigen Heterogeneity and Downregulation

This article reviews the development of antibody–drug conjugates [ADCs] as a game-changing modality in breast cancer treatment, using monoclonal antibodies to deliver powerful cytotoxic payloads directly to tumour cells, and discusses resistance mechanisms crosstalk and biomarker hurdles that are complicating but vital for patient selection, therapy sequence decision-making, and design of next-generation agents [86]. One of the primary mechanisms of resistance is due to antigen heterogeneity occurring within and between tumours, whereby differential or patchy expression of the target antigen—be it HER2, Trop-2, Nectin-4, or others—leads to non-uniform ADC binding and incomplete payload delivery; intratumoural heterogeneity may create regions containing low or absent levels of antigen that escape initial therapy and act as a source for disease recurrence while intertumoural heterogeneity makes treatment in metastatic settings challenging because lesions can markedly differ in terms of tissue expression targets, thereby making systemic ADC therapy give variable efficacy across disease sites [87]. A closely related phenomenon is when cells downregulate antigens, where tumour cells actively reduce or internalize expression of the ADC target as an adaptive response to therapy sometimes mediated by epigenetic changes, transcriptional suppression, or enhanced receptor endocytosis and degradation. This adaptation downregulation reduces binding and uptake of ADCs, thus limiting intracellular payload release and contributing to acquired resistance which has been observed in patients progressing on both first-in-class ADCs (e.g., T-DM1) as well as next-generation agents (e.g., T-DXd).

Other intracellular processes further complicate the issue, such as changes in endosomal trafficking diverting ADCs from lysosomal processing, drug efflux by occurring overexpression of ABC transporters, payload degrading enzymes, and induction of alternate survival pathways reducing apoptosis triggered by cytotoxicity [88]. These resistance mechanisms highlight the necessity for dynamic and robust biomarker approaches as static assessments of antigen expression by IHC or in situ at a single time point may be insufficient to capture temporal changes and spatial heterogeneity, misclassifying patients with potential implications for therapeutic failure. Liquid biopsy techniques specifically circulating tumour DNA and circulating tumour cells were actively investigated to afford real-time, minimally invasive assessment of how antigen expression, mutational landscape, and resistance mechanisms change over time as assays that rely on imaging with now evolved through implementation of radiation-modified antibodies or guided by near-ADC attempt data for target density and distribution across the body [89].

Clinically, there are practical implications of the relationship between antigen heterogeneity, downregulation, and resistance: patients with heterogeneous HER2 expression may initially respond in part to T-DXd due to its cleavable linker and mass effect but continued outgrowth of antigen-negative clones might eventually mediate disease progression; consequently, Trop-2-targeted ADCs such as sacituzumabgovitecan will have diminished activity in tumours with variable or low levels of Trop-2, emphasizing patient selection strategies based on threshold criteria and iterative therapeutic approaches [67]. From the translational point-of-view, the approach to next-generation ADC design aims at bypassing these limitations by employing several innovative strategies: biparatopic (bispecific) antibodies that bind multiple epitopes and recognize multiple antigens to enhance binding avidity in order to overcome partial loss of antigen expression on tumour cells, probody [90].

ADCs processed by proteases selectively present in the vicinity of the tumour microenvironment, thus limiting exposure outside, payloads with detrimental activity capable of diffusing into nearby “antigen low” cells, site-specific conjugation techniques improving pharmacokinetics and promoting better penetration across tumours from molecules with customized size and molecular structure isomers, combined approaches pairing up ADCs with immunotherapy or kinase inhibitors targeting a resistant subclones [91].

### 5.2. Efflux Pumps and Payload Resistance

Among these are P-glycoprotein (MDR1/ABCB1), MRP1 (ABCC1), and BCRP (ABCG2), although most transporters belong to the ATP-binding cassette (ABC) manufacturer family [92]. They serve to efflux DNA-damaging agents out of cytoplasm of hit cells, decreasing intracellular level to sublethal doses and thus attenuating ADC-related cytotoxicity. Data in pre-clinical and clinical environments have shown that cancer cells may over-express efflux pumps either constitutively or after ADC exposure, leading to resistance development which is also known to occur as collateral cross-resistance with other chemotherapies. This model is especially appropriate for the class of payloads auristatins, maytansinoids, and topoisomerase I inhibitors that are substrates of the transporters and at the same time very potent [93].

Further payload has inherent resistance mechanisms that may result in a multi-complex resistant landscape which will further reduce the potential for the piece to evoke cytotoxicity while achieving effects of delivery of payload is maintained. These targets include modifications of gene products (e.g., mutations in tubulin for microtubule inhibitors, topoisomerase I genes for exatecan derivatives), elevated repair pathways, and activation of anti-apoptotic signalling cascades [94]. Thus, ADCs with next-generation entities with payloads that are not good substrates for ABC transporters are further improved by site-selective conjugation, use of cleavable linkers, or detrimental effects abating intracellular heterogeneity in drug accumulation, as well as the co-administration of MDR inhibitors (i.e., combined therapy), for which we have an extended strategy under investigation at present that has experienced little clinical translation to date due to reasons related to both toxicity and pharmacokinetics interaction [95]. In addition, “moiety-based” combination approaches involving ADCs plus immune therapies or survival pathway antagonists (e.g., PARP inhibitors, BCL-2 family antagonists) are being pursued to sensitize resistant tumour subpopulations and mitigate efflux-mediated as well as payload-intrinsic resistance [96].

### 5.3. Tumour Microenvironment Barriers

ADCs have transformed precision medicine for breast cancer by permitting highly potent cytotoxic drugs to be specifically directed towards tumour cells. Nevertheless, the TME as a physical and biochemical obstacle to ADC activity is receiving more attention and affecting the clinical effectiveness of ADCs. This represents a complex and multiform resistance mechanism, which complicates drug response and biomarker-based patient selection [97]. Few TME components show a profile of activity including cytotoxic cell accessibility, APC function, abrogation of the PD-1 axis and modification co-regulators, and surface antigen presentation beyond LC50 that impact ADC uptake, distribution, and anti-target payload effect. In breast tumours, intratumoural distribution of ADCs is uneven, possibly due to physical blockade by fibrotic ECM deposition and dense desmoplastic stroma. This indicates that, although hypoxic- or poorly vascularised-core regions are still underexposed, subpopulations resistant to the drug du jour do persist at peripheral tumour sites [98].

Abnormal tumour vasculature also potentiates IT drug heterogeneity, which is induced by uneven vessel diameters, gradient of arteriole size distribution, heterogeneous perfusion, and high interstitial fluid pressure (IFP); thus, it hinders even ADC extravasation and delivery throughout the tumours. The TME facilitates resistance in an active manner through both cellular and molecular mechanisms, aside from these physical barriers. The sensitivity of ADCs can be reduced by tumour-associated fibroblasts (TAFs) that release growth factors, cytokines, extracellular vesicles capable to stimulate a process similar to epithelial–mesenchymal transition, enhance survival signals, and target antigen expression modulation [99]. Tumour-infiltrating macrophages may be able to take up ADCs or release cytotoxin and act by removing it from the tumour microenvironment but they also secrete immunosuppressive cytokines, like TGFβ with suppressed antigen-specific priming that downmodulate cell death [99].

Two advantages garnered from hypoxia to the tumour cells due to such responses in TME adaptative changes are decreased potency of intracellular payload and increased clonal survival under sublethal exposure to ADCs. These responses involve upregulation of efflux transporters, stimulation of the expression of DNA repair pathways, and metabolic adaptations. In addition, the acidic pH of TME as well as proteases and ROS may impact on activity of payloads or destabilize linkers that may reduce therapeutic potency or side effects [100]. Instead, new methods such as multiplexed spatial transcriptomics, advanced imaging, and functional assays of drug distribution aim to reflect the heterogeneity of TME, perfusion status, and stromal content to optimize patient stratification [101].

### 5.4. Predictive Biomarkers and Companion Diagnostics

Strategies for development and incorporation of predictive biomarkers offer both promise and challenges in the era of precision oncology. ADCs have revolutionized the treatment of breast cancer through targeted delivery of cytotoxic payloads. In the clinical setting, these are highly dependent on companion diagnostics and predictors of benefit that are able to select for patients who would most likely benefit from strategies targeting these cancers as well as predict those who are responding with an appropriate treatment and subset potential emerging resistances [102]. For two ADCs, trastuzumab-emtansine (T-DM1) and trastuzumab deruxtecan (T-DXd), patient selection based on traditional biomarkers such as expression of HER2 by immunohistochemistry (IHC) or in situ hybridization has played a role for HER2-positive breast cancer. However, these fixed time-point, single-level assessments often fail to account for intratumour heterogeneity, temporal variations in antigen density, and the presence of resistant subclones leading to potentially suboptimal treatment planning [101].

Blood-based biomarkers, including extracellular vesicles, circulating tumour cells (CTCs), and circulating tumour DNA (ctDNA), offer non-invasive procedures to obtain a real-time assessment of treatment efficacy, quantitate shedding of target antigen, or identify emerging resistance mutations and track tumour development over time. These biomarkers could also be of use as indicators in relation with adaptive therapy and early intervention strategies. These are increasingly applied in companion diagnostics, combining genomic, proteomic, and functional readouts to predict the likelihood of response, stratify patients for ADC therapy, and inform combination strategies with kinase inhibitors, immune checkpoint inhibitors, or other targeted agents [103]. Predictive biomarkers are also important to direct toxicity and safety. For instance, they can be used to identify patients at high risk of developing neutropenia with sacituzumabgovitecan or interstitial lung disease with T-DXd to prepare for active monitoring, dose reductions, and supportive care interventions. New biomarker paradigms are still being explored through translational research including machine-learning models integrating multi-omic and clinical data to derive predictive models of response of ADCs and imaging-based diagnostics utilizing radiolabeled antibodies for in vivo visualization of target expression and distribution [104]. Patient selection remains paramount to harnessing the full potential of the antibody–drug conjugate and limiting off-target toxicity. In addition to widely accepted tissue-based assays like immunohistochemistry and in situ hybridization, several promising, dynamic, and minimally invasive biomarker modalities continue to be evaluated in the clinic. First, circulating tumour DNA allows for real-time tracking of tumour burden and molecular evolution and thus can be used to identify further antigen heterogeneity, resistant mutations, and other consequences of treatment. For instance, quantitative ctDNA tracking was shown to correlate with early progression in ADC-treated patients and could perhaps help support adaptive decision-making in therapy. Another example where circulating cells can be of an interest is looking for circulating tumour cells and surveying them, as a population, for potential antigen expression loss, activation, or modulation which represents a common. Use of CTCs alongside single-cell sequencing enables unprecedented precision in detecting the right patients for target-specific drugs. Third, imaging-based biomarkers enabled by the use of PET tracers coupled to antibody fragments, the payloads themselves, and other substance can be used to visualize time-course antigen distribution, drug uptake, and payload delivery. One way or another, use of the above six biomarker systems should, together or separately, support a transition towards fully data-driven ADC therapies with molecular and other indicators used to select both a cohort of patients and then monitor and, if necessary, adjust their therapy.

## 6. Beyond ADCs: Expanding the Targeted Therapy Arsenal

### 6.1. Bispecific Antibodies and Immune Cell Engagers

Bispecific antibodies (BsAbs) and immune cell engagers (ICEs) are two frontline proofs of concept for the next generation, which is quickly replacing ADCs as established drug modality classes in the fight against breast cancer. These approaches are designed to optimize the selectivity and immune activity of tumour-targeting antigens and effector mechanisms, engage resistance mechanisms, escape regulatory barriers or prednisolone shot costoroidal steroids that can block stage for ado-trastuzumab emtansine (HEE-tabu-siz-MAINE) adverse events as well [105]. Bispecific antibodies’ common strategy to increase binding avidity, enhance tumour penetration, or bypass limitations of heterogeneous target expression that may reduce the potency of ADCs is to develop bispecific antibodies recognizing two different epitopes on either the same antigen (biparatopic) or distinct antigens [105]. For instance, biparatopic HER2-targeted BsAbs are able to retain high-affinity engagement even in heterogeneous tumours expressing a broad range of HER2, enabling more potent receptor internalization and effective inhibition of the signal pathways or payload deposition when combined with ADC payloads or used as single cytotoxic effects [104].

Immune cell engagers, including bispecific T-cell engagers (BiTEs) and NK-cell engagers, engage cytotoxic effector cells to tumour cells through simultaneous engagement of a tumour-associated antigen and a receptor on effector immune cells (e.g., CD3 on T cells or CD16 on natural killer cells) without the requirement for toxic internalization or intracellular payload release observed with conventional ADCs [105]. This approach takes advantage of the host’s immune system to achieve localized cytotoxic effects which could potentially treat low- or heterogeneous-antigen-density tumours that are less likely to benefit from monotherapy with ADCs. The application of these strategies looks particularly promising in HER2-low and TNBC populations, where the heterogeneity of antigen expression and paucity of available treatments has typically limited therapeutic efficacy [106].

Both BsAbs and ICEs can potentially complement existing ADC platforms and provide combinatorial attack options based on immune-mediated tumour eradication as well as direct cytotoxicity about function. In several preclinical models such combinations have been demonstrated to enhance response rate and duration, prevent the emergence of resistant subclones, and potentially convert immunologically “cold” tumours into more inflamed, immune-reactive phenotypes. As immune effector engagement is less dependent on intracellular drug accumulation, it can target both antigen-positive and adjacent-antigen low tumour cells through immune cytotoxicity. The development of these next-generation ADC platforms are being designed to enhance tumour specificity, overcome antigen heterogeneity, reduce off-target toxicity, and improve clinical efficacy across diverse breast cancer subtypes, including antigen downregulation, efflux-mediated payload resistance, and tumour microenvironmental barriers. Both BsAbs and ICEs have shown promising activity in Phase I trials for breast cancer [107].

### 6.2. Antibody–Radionuclide Conjugates

Unlike traditional chemotherapeutic warheads, antibody–radionuclide conjugates that carry the monoclonal antibody component play a critical role in determining antigen specificity, internalization rate, and overall ADCs, pharmacodynamics, and a radioactive isotope (a β-emitter like lutetium-177 or an α-emitter like actinium-225 or astatine-211) to the tumour cells that express a specific target antigen. It allows for regionalised DNA damage and adverse resulted in death of tumour cells possible through different pathways as those that are permissive to immunogenic cell death, along with detrimental effect of surrounding antigen-low or -negative tumour cells [108]. Furthermore, high LET of α-emitters has the benefit of high cytotoxicity in a short range with less toxicity to surrounding normal tissue and off-target damage. This is favourable, particularly for diffuse micrometastatic disease or breast cancer that is adjacent to vital structures. ARCs may be able to induce cell death because of radiation-induced DNA damage even when the antigen-mediated internalization is not very efficient, implying that this approach targets a resistance mechanism within ADCs, which rely on internalization and intracellular trafficking for releasing their payload [109].

Preliminary phase trials have shown prolonged-arm activity in heavily pretreated patients, including those with lesions refractory to standard ADCs, and they tap into the potential for ARCs to potentially overcome both innate and acquired resistance. These are in clinical trials as ARCs for HER2-positive, low-HER2, and TNBC models [104]. Antibody affinity, antigen density, stability of linkers, and the radionuclide half-life are major variables that play a role in effecting the pharmacokinetics profiles and biodistribution of ARCs. All these parameters ultimately determine tumour penetration, retention, and off-target exposure; site-specific conjugation chemistry, engineered antibody formats, and cleavable linkers contribute to increased tumour targeting relative to systemic radiation exposure resulting in a higher therapeutic index [110].

### 6.3. Nanoparticle-Based Targeted Delivery

Nanoparticles can be programmed to house chemotherapies, RNA-based treatments, or imaging agents. They can be functionalised with tumour-targeting ligands (e.g., antibodies, peptides, aptamers, or small molecules) for selective uptake into the tumour via active targeting and using also the enhanced permeability and retention (EPR) effect for passive localization into tumours. Nanoparticles are constructed of a variety of materials, such as liposomes, polymeric nanoparticles, dendrimers, gold- or silica-related nanostructures, and exosome-mimetic vesicles [111]. By providing payloads not just to antigen-high cells but, via diffusion or detrimental effects, to tumour space surrounding low-expressing target, this dual-targeting approach circumvents survival of resistant sub-clones which often underlie the loss of ADC efficacy. This strategy addresses the widespread challenge of intratumoural heterogeneity.

Apart from conventional chemotherapeutics, nanoparticles could be engineered to co-deliver multiple drugs at the same time with/to immune modulators, siRNAs, miRNAs, or drugs that can cause synergism. This allows for targeting heterologous pathways such as immune evasion, apoptotic control, and DNA repair, which might increase the length of response upon treatment and concomitantly reduce resistance rates [112]. Indeed, ADC mimetics in the form of nanoparticle (NP)-based delivery systems such as Ab-conjugated nanocarriers can potentially mimic the specificity of ADCs and offer enhanced payload carrying capacity, drug modularity for incorporation, and imaging reporters for theranostic purposes. These mimetics also provide the opportunity for on-the-fly tumour-targeting, biodistribution, and therapeutic response profiling. Moreover, nanoparticles are an excellent platform for more advanced and combinational strategies, such as co-loading ADC payload together with radionuclide or immunomodulatory agents. This essentially merges multiple therapeutic platforms within a modular drug delivery system capable of circumventing both acquired and inherent resistance mechanisms [113].

### 6.4. Peptide–Drug Conjugates (PDCs) and Aptamer Platforms

ADC-based platforms prove limited moving forward, and alternative modalities such as PDCs and aptamers provide advantages for tumour specificity, tissue penetration, and modularity; the hunt for precision-targeted therapies in breast cancer is slowly but surely stepping beyond older technologies. It increases the therapeutic window and overcomes some of the limitations that are challenge-associated with conventional ADCs, such as target heterogeneity, microenvironmental barrier, and payload resistance [114]. In an effort to deliver cytotoxic payloads more accurately in a package that has a lower molecular size than native antibodies, specific targets are engaged by peptide–drug conjugates using short peptides with high target affinity for tumour surface receptors or antigens, many of which are overexpressed in breast cancer subtypes including HER2, EGFR, integrins, or other tumour-related surface proteins. For that reason, here, mAb has been treated with a protease for better tissue penetration and quicker tumour targeting together with ideal lowered immunogenicity. Although the peptide sequences and linkers can be tuned for stability, receptor selectivity, and controlled delivery of payload (e.g., typical chemotherapeutics, microtubule inhibitors, or DNA-damaging agents), PDCs also offer a way to reach densely fibrotic or poorly vascularised tumour regions that may not easily be penetrated by large ADCs [115].

Formed of short, single-stranded nucleic acids or oligonucleotides modified to change folding into distinct three-dimensional structures, aptamer-based systems bind tumour-associated proteins, cell surface markers, or elements in the extracellular matrix with exceptional affinity and selectivity at high density, providing a complementary approach to targeting; its small size also provides for more flexible design based on a biologically relevant yet compact antibody-like mechanism of action. In comparison to bio-derived antibodies, aptamer–drug conjugates can be tuned for rapid internalization and intracellular payload release while offering versatile application potential such as imaging, theranostics, or co-delivery of chemotherapeutics and nucleic acids [116].

The modular nature of PDCs and aptamer drug platforms enables the co-delivery of multiple payloads, immune modulators, or microenvironment-targeting agents, as well as combination therapies. This would overcome tumour heterogeneity, efflux-mediated resistance, and adaptive survival responses that limit the efficacy of ADCs. Such modalities have known advantages, in terms of a translational usage as well, such as a reduced systemic exposure and potential oral or subcutaneous administration and favourable pharmacokinetics by reasonable modifications (e.g., PEGylation, cyclization, addition of non-natural amino acids/nucleotides to improve stability even enzymatic resistance) [117]. Taken together, PDCs and aptamer-based systems are a major breakthrough in the repertoire of therapies targeting breast cancer. They provide even smaller, more selective, and manageable delivery systems capable of infiltrating challenging tumour microenvironments, bypassing widespread resistance mechanisms, plugging into multimodal therapeutic strategies, and facilitating a customized treatment with next-generation targeted therapy that is not only better but also better modifiable than conventional ADCs across multiple breast cancer subtypes [118].

## 7. Combination Strategies and Synergistic Approaches

### 7.1. ADCs with Immune Checkpoint Inhibitors

ADCs deliver highly potent cytotoxic payloads directly to the tumour cells resulting in direct cell apoptosis and immunogenic cell death (ICD), with the release of proinflammatory cytokines, tumour antigens, and damage-associated molecular patterns (DAMPs) that can attract and activate T lymphocytes, dendritic cells, and other effector immune populations in the TME [119]. This immunogenic cascade allows ICIs, including PD-1/PD-L1 inhibitors or CTLA-4 blockers, to strengthen antitumour immune responses by lifting inhibitory breaks in T cells, promoting the proliferation of cytotoxic T-cell populations, and increasing recognition and clearance of both antigen-positive and -negative tumour subclones [119].

It has also been demonstrated in preclinical models that the combination of ADCs and ICIs can convert an “immune cold” breast tumour microenvironment into a more inflammatory, T-cell-rich milieu. This increases tumour infiltration, cytokine production, and antigen presentation, leading to a potential solution for some of the limitations observed for single-agent ADC in particular tumours with low baseline immune activity or heterogeneous target expression [120]. As predictive factors including PD-L1 expression, tumour-infiltrating lymphocyte (TIL) density, tumour mutational burden, heterogeneity in antigen expression, and markers of immunogenic cell death can inform both the eligibility as well as potential benefit from such a combined therapeutic approach, translational studies underline the relevance of biomarker-guided patient inclusion into this combinatorial treatment scenario. It is due to the potential overlapping toxicities, such as irAEs or myelosuppression worsening, that safety management with ADC–ICI associations require careful monitoring [121]. If a favourable therapeutic index is to be maintained, then measures such as dose optimization, staggered rhythmicity, and vigilant monitoring are required.

Mechanistic rationales for triple-combination or multimodal strategies extend beyond single-agent synergy. In these strategies, ADCs elicit immune priming and tumour cell death, while ICIs relieve adaptive immune inhibition, and other targeted agents (e.g., kinase inhibitors, PARP inhibitors, or anti-angiogenics) influence parallel tumour survival pathways that can enhance efficacy by circumventing potential resistance mediated by payload efflux or tumour microenvironmental barriers [122]. From a translational perspective and into the near future, the development of ADCs in combination with ICIs represents an intellectual and practical maturation of targeting therapy that transcends cytotoxic, payload-centric approaches to embrace a fully integrated, immune-enhanced multi-mechanistic approach to treatment that has potential to enable better and durable responses for difficult-to-treat tumours, broaden precision application across subtypes of breast cancer such as HER2-positive disease and also new opportunities to treat HER2-low positive HR-positive or triple-negative breast cancer [120].

Combining ADCs with other therapies can help mitigate toxicity by allowing lower ADC doses while maintaining efficacy, thereby reducing exposure-related side effects such as neutropenia or gastrointestinal issues. For example, pairing ADCs with endocrine therapy in hormone receptor-positive breast cancer or with immunotherapy in triple-negative disease has shown synergistic activity, enabling dose de-escalation and schedule adjustments. Early clinical studies report that these combinations are generally manageable in terms of safety, though careful monitoring is required to avoid additive toxicities such as immune-related adverse events when checkpoint inhibitors are involved.

### 7.2. ADC and PARP Inhibitors

PARP inhibitors combined with ADCs are a novel and promising approach for treatment of mammary carcinoma. It is aimed at antitumour activity, and it is how resistant (intrinsic and acquired) resistance as well as adding on to it with a complementary mechanism of tumour cell death—particularly in DNA repair-deficient subtypes such as BRCA-mutated or HRD-containing tumours. ADCs transport highly cytotoxic payloads—most commonly microtubule-disrupting agents or a topoisomerase I inhibitor—specifically to cancer cells, leading to the infolding of DNA damage, cell cycle arrest, and apoptosis [123]. Tumour cells, in particular those with pre-existing deficiencies in the homologous recombination repair pathways, may fail to recover from combination of replication stress and DNA double strand breaks compartmentalized cytotoxicity. PARP inhibitors such as olaparib, talazoparib, and niraparib prevent the repair of single-strand DNA breaks. These accumulating DNA insults lead to replication fork breakdown, which generates synthetic lethality in HR-deficient cells [123].

Preclinical research suggests that the combination of PARP inhibitors and ADCs lead to an enhanced apoptotic effect, with a reduction in clonogenic survival and resistance induced by either therapy alone. This is particularly evident in TNBC and HER2-low or HER2-positive cancer models with deficiencies in DNA damage response [124]. It is germane because, even though PARP inhibitors drive cytotoxicity through a mechanism that does not depend on the action of antibody internalisers and can also achieve activity in neighbouring or low-antigen tumours via increased DNA damage-mediated susceptibilities, combinatorial schemes between ADC and PARP inhibitors may mean some extra few holes for resistant elements usually associated with ADCs, as a result of payload efflux, divergent antigen expression, or stromal obstruction [125].

Translational implications: Similarly to all cancer therapy, biomarkers are the critical clinical devices like those in enrichment for patients most likely to benefit maximally from this combined therapeutic strategy without subjecting potential non-responders to significant toxicities [126]. Phase I studies are ongoing to assess safety and efficacy in the combination of ADC–PARP inhibitors, considering overlapping toxicity including myelosuppression and gastrointestinal toxicities as well as potentially additive DNA damage [127]. Strategies such as dose, timing, and schedule interruption are being investigated to optimize their input, by enhancing the mixture of synergistic antitumour effects and minimizing impact on therapeutic index.

### 7.3. Hormonal Therapy and ADC Integration

Due to the precision-based cytotoxic nature of ADCs and tumour growth-suppressive effects consequential from endocrine modulation, concurrent use of an ADC and hormonal therapy is a promising new strategy for breast cancer treatment, particularly for hormone receptor-positive (HR+) subtypes. This combined modality strategy provides a multimechanistic philosophy leading to enhanced antitumour effectiveness, resistance avoidance, and heterogeneous tumour biology coverage [127]. Inhibiting estrogen receptor (ER) signalling with hormonal therapies including selective estrogen receptor modulators (SERMs; such as tamoxifen), aromatase inhibitors (such as letrozole or anastrozole), or selective estrogen receptor degraders (SERDs; such as fulvestrant) leads to cell-cycle arrest, decreased proliferation, and—in some cases—sensitisation to cytotoxic stress. Through antigen-driven internalization, the ADCs deliver highly potent cytotoxic payloads to tumour cells specifically and induce DNA damage, apoptosis, and detrimental effect (eg, T-DXd or sacituzumabgovitecan). This is particularly valuable in tumours expressing target heterogeneously [128].

To maximally align both arms of therapy with tumour biology, biomarkers such as ER/PR status, HER2-low expression, Trop-2 density, and proliferation indices (Ki-67), along with genomic alterations within endocrine resistance pathways, can be employed to direct dosing schedules and guide patient populations. To improve PFS, maximize response rate, and extend DOR, trials are now evaluating ADCs in combination with aromatase inhibitors (AIs), SERDs, or CDK4/6 inhibitors in mBC HR+/HER2-low. These studies are also watching for overlapping toxicities such as neutropenia, gastrointestinal events, and potential liver or pulmonary cumulative effects. This combination strategy might also be potentially enhanced by combining with other targeted agents such as PI3K inhibitors, PARP inhibitors, or even immune checkpoint blockade that exploit synthetic lethality, immune priming, and pathway inhibition to maximise tumour cell kill while preventing adaptive resistance [129].

Taken together, hormonal therapy and ADCs offer a rational, biomarker-driven approach to the next-generation treatment of breast cancer in conjunction with endocrine modulation and targeted cytotoxicity. This strategy combats tumour heterogeneity, resistance mechanisms, and depth/duration of response while extending the spectrum of treatments to encompass HR+ and HER2-low disease, paving the way for personalized, multi-modal, and adaptive therapies in early-stage as well as metastatic settings [25].

## 8. Safety, Toxicity, and Management

### 8.1. Hematologic and Gastrointestinal Toxicities

Systemic exposure of the rapidly proliferating BM progenitor cells to ADC payloads, including topoisomerase I inhibitors and microtubule-disrupting agents (such as SN-38 or MMAE) that can circumvent targeted tumour saturation by residual blood flow or non-specific uptake, appears to be largely responsible for hematologic toxicities (e.g., neutropenia, anemia, thrombocytopenia, and, less frequently, leucopenia). MMAE and MMAF are extremely potent tubulin inhibitors and their toxicity can be mitigated through several design strategies in antibody–drug conjugates. Using stable, tumour-selective linkers ensures that the payload is released only inside cancer cells, while site-specific conjugation and optimized drug-to-antibody ratios reduce off-target exposure. Hydrophilic spacers and controlled-release linkers improve solubility and pharmacokinetics, lowering systemic toxicity. Additionally, MMAF, with its charged structure, is less membrane-permeable than MMAE, limiting diffusion into normal tissues. Careful antigen selection, dosing optimization, and supportive clinical measures further enhance safety while maintaining therapeutic efficacy.

High grades of neutropenia as dose-limiting toxicity were reported in several clinical trials of ADCs, including further applied sacituzumabgovitecan and trastuzumab deruxtecan [130]. Thus, it is required to perform strict blood count control, as well as prophylactic G-CSF administration, and dose adaptation or cessation of treatment when the thresholds are achieved. Neutropenia is particularly worrisome for the risk of febrile and opportunistic episodes [130].

Although often milder, but with no less quality-of-life impairment, anemia and thrombocytopenia might require supporting measures like dose reductions, use of iron, or blood transfusions. Off-target cytotoxic effects of ADC payloads on rapidly dividing mucosal epithelial cells lining the gastrointestinal tract and enterohepatic recirculation of payload metabolites drive one spectrum of GI toxicities that include nausea, vomiting, diarrhea, and mucositis with more severe presentations rarely observed such as colitis or hepatotoxicity [131]. Antidiarrheal drugs including loperamide, hydration assistance, and patient education to recognize early symptoms are required because diarrhea is particularly frequent in ADCs containing a topoisomerase I inhibitor with the potential for dehydration, electrolyte imbalance, and treatment interruption in untreated patients.

There are several factors that influence the extent of hematologic and GI toxicity, including ADC payload potency, linker stability, antibody specificity, dosing schedule, prior therapy, and individual patient characteristics such as baseline marrow reserve or co-morbid conditions. Clinic integration in terms of multidisciplinary care, detection at an early stage, and compliance to evidence-based supportive interventions (e.g., blood count monitoring rates, prophylactic growth factor support, antiemetic prophylaxis, and patient education for self-monitoring of gastrointestinal symptoms) is inherently key to a proactive form of toxicity management [132].

### 8.2. Interstitial Lung Disease and Cardiotoxicity

Incidence rates have been reported at 5% to 15% in clinical trial as well as real-world cohorts and ICI-ILD, most observed with HER2-targeted antibody–drug conjugates such as trastuzumab deruxtecan (T-DXd), may present on a spectrum of radiographically asymptomatic findings to severe, life-threatening pneumonitis and respiratory failure. ADC-related ILD is a multifactorial condition involving immunomediated inflammation, off-target delivery of the cytotoxin payload by alveolar epithelial cells, and, in some cases, preexisting lung damage worsened by prior thoracic radiotherapy or pneumopathy [133]. These patients can show up with nonspecific symptoms such as cough, fatigue, low-grade fever, or dyspnea and a significant proportion of their disease burden is overestimated if not prospectively followed on imaging. Early intervention is needed to prevent irreversible pulmonary fibrosis. It is managed by early diagnosis with HRCT, cessation, or discontinuation of ADC therapy, corticosteroid treatment in patients with moderate to severe manifestations, and supportive care of the respiratory system [134].

### 8.3. Strategies for Dose Optimization and Monitoring

The dichotomy of an ADC as both a biologic targeting moiety and a highly cytotoxic payload leads to inherently complex dose optimization approaches. Several factors contribute to the safety and efficacy of ADCs, including patient pharmacokinetics, stability of delivery vehicle (linkers), potency of payload, affinity of Ab-antigen binding, and DAR [132]. Empirical and clinical data have taught us that dose selection is often dictated by carefully considered estimates of the maximum tolerated dose (MTD), starting low to strike a balance between safety and efficacy. This is particularly pertinent for individuals who have previously received cytotoxic chemotherapy, radiotherapy, or other targeted agents that could compromise marrow reserve/organ function [135]. Although in its infancy, therapeutic drug monitoring is increasingly being used to quantify variability in the ADME (absorption, distribution, metabolism, and excretion) of ADC payload exposure and plasma concentrations. This enables personalized dosing and an early response to systemic concentration exceeding the safety thresholds. Concomitantly, for achieving the maximum possible tolerability, the dose-finding and -adjusting measures are enriched with patient-individual factors like age, presence of comorbidities, organ function, or co-medication. The multitiered monitoring strategies include pulmonary imaging and symptom screen for interstitial lung disease; assessment of liver and renal function tests to detect subclinical organ toxicity; echocardiography and cardiac biomarkers vigilance for the cardiotoxicity; and serial haematologic exams screening the neutropenia, anemia, and thrombocytopenia [136].

## 9. Translational and Manufacturing Perspectives

Preclinical models are the basic platforms required to fill a huge gap between molecular jokes and patients’ needs in designing, optimizing, and translational advancing of ADCs into breast cancer. Such models are indispensable for understanding the mechanism of action, evaluating efficacy and toxicity, and guiding clinical trial design [137]. To mimic breast tumour biology and the intra-tumoural microenvironment, these systems include an array of in vitro and in vivo models including organoids, patient-derived xenografts (PDXs), syngeneic tumour models, genetically engineered mouse models which develop spontaneous or orthotopic lesions, humanized mouse platforms, as well as two-dimensional (2D) and three-dimensional (3D) cell cultures. They both have their own pros and cons [138].

In vitro studies can be applied in initial optimization of the antibody selection, the linker chemistry and payload (i.e., cytotoxic agent) determination for rapid high-throughput evaluation of ADC binding specificity, internalization kinetics, optimal cell lysis potential, as well as resistance mechanism to treatment including efflux pump activity, DNA repair ability, and antigen heterogeneity [139]. Three-dimensional culture systems and organoids are more representative of the tumour, with respect to architecture, cell heterogeneity, ECM interactions, and drug penetration dynamics, which all influence ADC distribution, detrimental effects, and efficacy, thus increasing translational relevance [139]. While humanized mouse models with patient-derived xenografts (PDXs) and genetically engineered mouse models (GEMMs) can help enable the study of immune-mediated effects, immune checkpoint biology, ADC interactions, and tumour microenvironmental interfaces, in vivo preclinical systems such as PDX and GEMM provide opportunities to gain an understanding of pharmacokinetics, biodistribution, tumour penetration, target engagement, systemic toxicity, or toxicology at a physiological level [140].

To ensure consistency and a high yield, the manufacture process begins with development of the monoclonal antibody through recombinant expression systems, such as Chinese hamster ovary (CHO) cells. This can be achieved with strict control of the parameters leading up to it, i.e., cell line stability, culture conditions, and protein expression levels before lysis. Downstream purification methodologies including protein A affinity chromatography, viral inactivation/removal, aggregation, or impurity removal are essential to obtain a product meeting stringent purity and safety needs [140]. These approaches, such as site-specific conjugation strategies, for example, can enhance batch-to-batch consistency, reduce off-target toxicity, and modulate therapeutic index via the control of payload distribution and reduction in unconjugated species. Both stochastic and site-specific payload conjugation represent a critical step impacting the direct drug-to-antibody ratio (DAR), linker stability, or general pharmacologic behaviour. Linker chemistry, the parameter that is responsible for controlling payload release kinetics and specificity, needs to be optimized rigorously to ensure stability during circulation and efficient intracellular release upon internalization within tumour cells. Predictive shelf-life and in vivo performance stability studies are performed over a full range of pH, temperature, and stress conditions [141].

## 10. Future Directions and Personalized Oncology

There are several ongoing and emerging trends and technologies that are shaping the next generation of antibody–drug conjugates. Despite the well-documented potency and clinical effectivity, the most therapeutic agents remain as highly toxic payloads with limited selectivity to the targeted tumour tissues with lower potency in the larger population. Immune-activating ADCs, for instance, combine cytotoxic payloads and immune-modulatory functions by promoting the activation of dendritic cells and recruitment of T-cells within the tumour environment. This dual modality has the potential to convert immunologically “cold” tumours into “hot” ones, thus rendering them more susceptible to checkpoint inhibitors and other immuno-therapeutic interventions. The second emerging concept is the dual-target or the bispecific ADCs that for the first time allow the engagement of two TARs within the cancer histospectrum. This will potentially overcome the resistance due to heterogeneity and reduce the chances of tumour neo-escape due to downregulation or loss of targeted antigen from active threats. Lastly, the in silico modelling, with the aid of artificial intelligence and machine learning, uses multi-omic data and allows patient selection through predictive ADC efficacy signatures, optimization of linkers and payloads, and the planning of adaptive trials. Overall, these emerging technologies and effector molecules will assist in the accelerated shift from the traditional highly toxic cytotoxic drugs to more potent, TAM-integrated, and selective therapeutics that have a lower sides toxicity and individual effects.

### 10.1. ADCs in Early-Stage and Neoadjuvant Settings

A move away from the previous roles in established metastatic and refractory disease, towards earlier intervention with goals of deeper and more durable responses, shrinking (operable) tumour load prior to surgery, as well as improved outlooks for any affected individuals through an approach blending precision oncology is conveyed by the focus on ADCs in early-stage and neoadjuvant contexts. This is a new era in the treatment of breast cancer [142]. As tumour tissue is available for biomarker analysis, pharmacodynamic evaluation, and to assess outcome of therapy, it creates a unique opportunity to utilize the neoadjuvant setting with ADCs in stages I–III breast cancer for both cytoreduction and biologic insight. These data instead guide later adjuvant therapies and personalized treatment schedules [143].

The application of imaging, circulating tumour DNA (ctDNA), tumour-infiltrating lymphocytes (TILs), and dynamic biomarker profiling as an approach to assess therapeutic efficacy in real time is also enabled by neoadjuvant ADCs. This facilitates adaptive therapy adjustments according to molecular features, early response, and the degree of residual disease [144]. Importantly, by destroying aggressive tumour clones prior to their capacity to seed metastatic niches, the use of early-stage ADCs has the potential to counter active resistance mechanisms observed in late stages of disease (e.g., antigen heterogeneity, efflux pump-mediated payload extrusion, or off-target barriers presented by intra-tumoural microenvironment). Synergy to maximize cytotoxicity, drive durable immunologic memory, and enhance long-term recurrence-free and overall survival is provided by integration with conventional neoadjuvant modalities including immune checkpoint inhibitors in TNBC, endocrine therapy in HR-positive disease, and chemotherapy for high-risk subtypes [145]. Given the potential for hematologic, gastrointestinal, pulmonary, and cardiac toxicities, safety and tolerability remain important considerations that will require careful patient selection, dose titration, and monitoring strategies accounting for cumulative exposure and combination therapy [146].

### 10.2. AI-Driven Target Discovery and Payload Design

Unprecedented precision in target identification, payload development, and therapeutic optimization is now possible with artificial intelligence (AI) and machine learning which have the potential to revolutionize the next generation of ADCs for breast cancer, accelerating the transition from empirical drug discovery towards a data-driven personalized oncology paradigm [147]. AI-driven target discovery uses extensive genomic, transcriptomic, proteomic, and single-cell multi-omics datasets to identify highly expressed tumour-specific antigens and neoantigens with little off-target expression and functional importance for tumour survival or progression including those overexpressed across breast cancer subtypes such as HER2-lo, TNBC, or Luminal B tumours. To predict which antigens are most likely to be accessible and internalized, and can support efficient ADC uptake, machine learning approaches can incorporate multi-omic data into the model such as mutation profiles, epigenetic alterations, spatial transcriptomics data, immune infiltrates, and tumour microenvironmental signals [148]. This makes antibody selection more directed and specific for the target. Through prediction of cytotoxic potency, modelling molecular interactions and optimizing linker chemistry for controlled tumour specific release with minimal systemic exposure and off-target toxicity, AI-driven platforms facilitate payload design as well as target identification [149]. Computational approaches may be used to simulate time and cost savings as well as reduced rates of attrition by predicting structure–activity relationships, drug–antibody conjugation efficiencies (DACE), pharmacokinetics, and tissue distribution. This would enable in silico screening of novel cytotoxic payloads, immune modulators, microtubule inhibitors, DNA damaging agents, and synthetic small molecules before experimental verification [150].

### 10.3. Patient Stratification and Adaptive Trial Designs

To characterize patients who are most likely to derive benefit from specific ADC constructs (e.g., HER2-directed, Trop-2-, or HER3-targeted therapies), patient selection requires the combination of a multi-dimensional approach that incorporates genomic alterations, transcriptomic signatures, proteomic profiles, tumour antigen expression levels, density of TILs, immune checkpoint status, and microenvironment characteristics. In that one, patients at increased risk for off-target toxicity or suboptimal response are precluded [151]. Moreover, advanced molecular profiling, such as next-generation sequencing (NGS) and single-cell RNA sequencing for determinants of response prediction or resistance mechanisms, as well as spatial transcriptomics and circulating tumour DNA (ctDNA) analyses can allow the dynamic characterization of tumour heterogeneity, clonal evolution, and adaptive resistance mechanism to select the optimal antibody, payload, linker chemistry, and combination strategy in individual patients [152].

Longitudinal surveillance by imaging, ctDNA, and functional assays is facilitated through adaptive approaches. This allows early assessment of tumour evolution, minimal residual disease, and emerging resistance that could lead to real-time therapy adjustment and patient-specific sequencing strategies. Adaptive, biomarker-selected trials promote efficient, mechanism-of-action informed evaluation while maintaining rigorous requirements of safety, performance, and quality [153]. They are also consistent with evolving regulatory requirements, which now favour patient-focused evidence, accelerated approval, and postmarketing confirmatory studies. In other words, patient stratification and adaptive trial designs combine to enable a flexible precision-led approach to the development of ADCs which can result in a personalized treatment that incorporates patient susceptibilities, tumour heterogeneity, and resistance mechanisms. It also strives for rapid optimization, strong translational insight, and further clinical usage [154]. Compelling, taken together, these approaches have the potential to transform the clinical landscape of breast cancer by allowing next-generation ADCs and targeted therapies to be delivered in a manner that is most effective with the least toxicity, while achieving personalization, adaptability, and mechanism-driven oncology strategy [155].

## 11. Limitations

Although considerable progress on a clinical scale was made, the current state of ADC development is limited by several factors impeding therapeutic consistency and broader clinical application. Most prominently, the complex array of resistance mechanisms hinders their effective use, results of which can differ significantly between biomarkers or even between targeted antibody–drug conjugates. Antigen downregulation, lysosomal dynamics, altered intracellular trafficking of the conjugates, and enhanced efflux of ADCs from the cells are the prevalent mechanisms of resistance. Furthermore, single biomarker reliance can limit the variety of applicable treatment scenarios due to the apparent unfeasibility of taking into account understandings of frequently disparate molecular compositions of the ordered tumours several years postdiagnosis.

## 12. Conclusions

ADCs, as a next generation of targeted therapies, have redefined the field of precision oncology and validated the intersectionality among subtypes including HER2-positive, HER2-low, hormone receptor-positive and -negative disease by marrying tumour-specific targeting with potent cytotoxic or immunomodulatory mechanisms to previously unimaginable levels of efficacy. ADCs represent a pinnacle of the convergence between as they deliver highly potent payloads selectively with minimal systemic toxicity through monoclonal antibodies. Their clinical success has also driven the way of new platforms such as peptide–drug conjugates, antibody–radionuclide conjugates, bispecific antibodies, and nanoparticle-based delivery systems that led to an explosion in the number of next-generation targeted therapies. The therapeutic potential of ADCs is additionally broadened through their evaluation in neoadjuvant and early phase, providing opportunities for tumour debulking, elimination of chemo-resistant clones, and combinations with multimodal therapy aimed at durable responses to ultimately improve overall survival. In the 21st century, new-generation targeted therapies can dramatically impact the lives of patients with breast cancer at every stage of progression from early to refractory/metastatic setting. With advance in research, construction of ADCs, combinations with other therapeutic modalities, and application of cutting-edge computational and biomarker methods will strengthen their position as mainstay therapies. Antibody–drug conjugates (ADCs) have transformed the therapeutic landscape of breast cancer by combining the target specificity of monoclonal antibodies with the potent cytotoxic activity of small-molecule payloads. Landmark clinical trials such as DESTINY-Breast03, ASCENT, and TROPION-Breast01 have demonstrated unprecedented improvements in progression-free and overall survival, establishing ADCs as a core component of modern breast cancer treatment across HER2-positive, triple-negative, and HR+/HER2− subtypes. Despite these successes, several challenges remain. Therapeutic resistance driven by antigen modulation, drug efflux mechanisms, and tumour heterogeneity continues to limit the durability of response. Furthermore, biomarker variability and the lack of standardized trial endpoints hinder cross-study comparison and optimal patient selection. Addressing these gaps will require integrated multi-omics profiling, dynamic biomarker monitoring, and harmonized clinical trial frameworks. Looking forward, the field is rapidly evolving toward next-generation ADCs that incorporate immune-stimulatory payloads, dual-target designs, and artificial intelligence-assisted biomarker discovery. These innovations promise to refine targeting precision, expand therapeutic windows, and personalize treatment strategies. In summary, ADCs represent a paradigm shift in breast cancer therapy—bridging molecular specificity with potent cytotoxicity. Continued interdisciplinary innovation will be essential to overcome resistance, enhance safety, and fully realize the potential of ADCs as cornerstones of precision oncology.

## Figures and Tables

**Table 3:** ADCs in Phase III clinical trials.

Name	NCT Number	Target	mAb	Linker		Payload	Phase
ARX-788		NCT05426486	HER2	IgG1	Hydroxylamine-PEG4	MMAF	Phase II/III
BNT323/DB-1303		NCT06265428, NCT0601833, NCT06340568	HER2	IgG1	mc-Gly-Gly-Phe-Gly	P1003	Phase III
DP303c		NCT06313086, NCT05901935	HER2	IgG1	PEG2-Val-Cit-PABC	MMAE	Phase III
FS-1502		NCT05755048	HER2	IgG1	Geranyl ketone pyrophosphate oxime ligation	MMAF	Phase III
TQB-2102		NCT06561607	HER2	IgG1	Undisclosed	Undisclosed	Phase III
Trastuzumab duocarmazine/SYD985		NCT03262935	HER2	IgG1	mc-PEG2-Val- Cit-PABA-Cyc	seco-DUBA	Phase III (completed)
SHR-A1811		NCT05814354, NCT06828354, NCT06057610, NCT06199973, NCT06430437, NCT06126640, NCT05424835, NCT06123494	HER2	IgG1	mc-Gly-Gly-Phe-Gly	SHR9265	Phase III
MRG-003		NCT05751512	EGFR	IgG1	mc-Val-Cit-PABC	MMAE	Phase III
MRG-002		NCT04924699, NCT05754853	HER2	IgG1	mc-Val-Cit-PABC	MMAE	Phase II/III
Zilovertamabvedotin		NCT06717347, NCT05139017	ROR1	IgG1	mc-Val-Cit-PABC	MMAE	Phase II/III
Depatuxizumabmafodotin/ABT-414		NCT02573324	EGFR	IgG1	Maleimidocaprol	MMAF	Phase III (completed)
Patritumabderuxtecan		NCT05338970	HER3	IgG1	mc-Gly-Gly-Phe-Gly	DXd	Phase III
FDA018		NCT06519370	TROP2	IgG1	Undisclosed	SN-38	Phase III

## Data Availability

The data presented in this study are available on request.
